# *History of a Case of Introsusception; and an Account of Two Cases of Stricture in the Urethra, Removed by Tobacco Bougies; in a Letter to Dr*. Redman Coxe, *of Philadelphia*

**Published:** 1807-10

**Authors:** William Shaw


### *History of a Case of Introsusception; and an Account of two Cases of Stricture in the Urethra, removed by Tobacco Bougies; in a Letter to Dr.* Redman Coxe, *of Philadelphia.* By William Shaw, M. D.

Dear Sir,

I HAND you the history of a very singular case of introsusception, which you may give a place in your Museum, if you should think it worthy of publication.

J. A. became a patient of mine some time ago; he had fever, with slight paralysis of his lower extremities; for which he was bled and had a mild cathartic given him, and this was repeated on the third day: his diet was ordered to be mild and nutritive. At the end of ten days he appeared so far recovered as to require no more medical assistance.

A few days after he was seized with diarrhœa; he did not make known his complaint until the seventh day. By this time he was very much reduced, but by the operation of a little castor oil, followed by a mild astringent medicine, his diarrhœa was stopped about the end of the third or fourth day, when his appetite began to return, and his strength seemingly to increase.

Four days had elapsed when the paralysis of his lower extremities returned, accompanied with sickness at his stomach, which sometimes amounted to vomiting; feeble and irregular pulse, with his countenance very much dejected; he said he felt as if his bowels were turning from one side of his body to the other, and sometimes they seemed to move from below upwards, so as to affect his breathing; all this time he complained of very severe pain. He was ordered a dose of laudanum (it being late in the evening): next morning he died. I opened the body soon after death; the contents of the thorax were in a healthy state: upon opening the abdomen, I found the stomach, duodenum, and about half of the jegunum very much distended with wind, the other part of the jegunum was filled, or nearly so, with more than half of the ilium next to it. At the commencement of this introsusception, a stricture existed, which nearly closed the intestine: three other strictures were found in the lower portion of ilium; and at each of those strictures a portion of intestine below, was received into that immediately above, one of about six, one of four, and a third of two inches. The whole of the small intestines showed a high degree of inflammation to have existed before death, and a part of the largest introsusception introsusception was already in a gangrenous state. The received portion of intestine was very readily withdrawn from the receiving.

Would not the surgeon be warranted in attempting an operation for the cure of this disease where the symptoms so plainly evinced its existence as in the above case? There can be but two material objections urged against the operation, viz. the fallacy of symptoms in diseases, and the fear of subsequent inflammation from exposure of the contents of the abdomen, during the operation, to the influence of the air. The first ought always to be a serious objection while any doubts remained of the presence of the disease; the second ought not to operate one minute as an objection, after the first was removed, and the common means proved ineffectual. We have many instances where, in consequence of wounds, the contents of the abdomen have been exposed for a length of time much greater than would be required for performing the proposed operation, and yet the persons have recovered. One of those dreadful cases came within my knowledge: the man received a wound in the abdomen by falling on a sharp instrument, which passed into the cavity, and let out a portion of intestine; he afterwards walked about three miles with the intestine in his hand, and by having the necessary means used, recovered in a reasonable time.

The following is the result of two experiments which I made with the nicotiana tabacum in the cure of strictures of the urethra. It is too well known to surgeons, that the disease in question often baffles every attempt to remove it; and I am of opinion that every fact relative to the cure of so painful and so distressing a disease should be made known. If publishing the history of those experiments should be the means of giving relief in a single instance, I shall feel myself amply rewarded for the time I have spent in copying them from my notes.

T. B. laboured under a stricture of the urethra seated near the prostrate gland about nine months. This disease owed its origin to an improperly treated gonorrhœa.

It would be too tedious to relate minutely all the different methods that were employed to remove it: Suffice it to say, that many trials were made with the common bougie; gum-elastic catheter, &c. blood-letting general and local, blistering on the perinæum, several courses of mercury, all the routine of diuretics, &c. and a spare diet. As a last resource the caustic was proposed by my worthy friend friend Dr. Physick; but previous to the trial with the caustic, I was induced, from having frequently evidenced the very powerfully relaxing effects of the nicotiana in the cure of spasmodic diseases, to make the following experiment, viz.

A small-sized bougie was procured, around which I wrapped a thin smooth leaf of strong tobacco, previously moistened with water, so as to enable me to give it a degree of smoothness, that it might with more ease pass into the urethra. My bougie being first moistened in a decoction of tobacco, was very gently introduced into the urethra down to the stricture, where it was kept moderately pressed against it for the space of about fifteen minutes, when it passed the stricture and went into the bladder: at this time my patient complained of sickness at his stomach, and although it was in the coldest season of the year, a considerable moisture appeared on his forehead; immediately after the bougie was withdrawn he discharged upwards of half a pint of urine, which flowed in a natural stream: the bougie was introduced twice afterwards the same day, and repeated daily for two or three days, without the least difficulty: he has continued free from the disease ever since.

The success I met with in the above case, led me to make trial of my bougie, with a small improvement, in a second and very similar case which occured to me a short time after.—This was an elderly man, who had by times suffered greatly from a stricture which existed about the commencement of the bulb of the urethra. This case had been treated in every respect similar to the foregoing, and with no better success. I made an extract of nicotiana and covered a small bougie with it, and having rendered it smooth, I moistened it in some of the same extract liquified by the addition of water: I made an attempt to introduce it into the urethra, which succeeded after a few minutes gentle pressure with the point against the membrane, which formed the stricture; a similar sensation of sickness and debility took place in this as in the other case, while the bougie was in the penis; the operation was repeated two or three times after this, which produced such torpor and relaxation of the sphincter vesicæ as to render him incapable of retaining his urine; however, this inconvenience was removed by taking a few drops of the tr. canthar. About six months after, I saw my patient, when he informed me that he had remained entirely free from every symptom of the disease during that time.

*Philadelphia, November* 1, 1805.

